# Hypercalcemia Secondary to Elevated PTHrP in an Infant Followed by Progression to Nephrotic Syndrome

**DOI:** 10.1210/jcemcr/luae074

**Published:** 2024-05-03

**Authors:** Alex F Gimeno, Tracy E Hunley, Jennifer C Kelley

**Affiliations:** Department of Pediatrics, Vanderbilt University School of Medicine, Nashville, TN 37232, USA; Department of Pediatrics, Division of Pediatric Nephrology, Vanderbilt University Medical Center, Nashville, TN 37232, USA; Department of Pediatrics, Division of Pediatric Endocrinology and Diabetes, Vanderbilt University Medical Center, Nashville, TN 37232, USA

**Keywords:** congenital nephrotic syndrome, parathyroid hormone, parathyroid hormone-related protein, hypercalcemia

## Abstract

In infants, hypercalcemia from elevated parathyroid hormone-related protein (PTHrP) is rare, often signaling neoplasm or renal or urinary anomalies. We report an infant who presented with failure to thrive and hypercalcemia at 10 months old, with initial evaluation showing elevated PTHrP of unclear etiology with imaging negative for neoplasm and no structural anomalies of the kidneys or ureters on ultrasound. Within 6 months of presentation, the patient developed nephrotic syndrome and by 2 years had progressed to end-stage kidney disease, necessitating kidney transplantation. Genetic testing was inconclusive but suggested congenital nephrotic syndrome. While reports of hypercalcemia secondary to elevated PTHrP exist in children with known structural renal anomalies, this is the first to demonstrate hypercalcemia and PTHrP elevation before detection of renal abnormalities. Experimental models have suggested a role for increased PTHrP expression in renal cells following acute kidney injury from nephrotic syndrome, and clinically detectable PTHrP levels may indicate progression of renal injury. We suggest monitoring of renal function for early detection of nephrotic syndrome in infants and children with elevated PTHrP who otherwise lack anatomical renal anomalies or detectable malignancies.

## Introduction

Severe hypercalcemia in infants is rare but clinically significant, and identifying the etiology is crucial given potential sequelae including failure to thrive, nephrocalcinosis, and seizures (1). The differential diagnosis for an infant with hypercalcemia is broad and can be divided into PTH-dependent and -independent categories. PTH-dependent causes include parathyroid hyperplasia and genetic causes of parathyroid overactivation, including hypercalcemic hypocalciuria (neonatal severe primary hyperparathyroidism), multiple endocrine neoplasia syndromes, McCune–Albright syndrome, and Jansen metaphyseal dysplasia. PTH-independent causes have been reported in cases of reduced renal function (such as post-renal transplantation, Barter syndrome, and renal tubular acidosis) and chronic hyperphosphatemia and situations of excess calcium or vitamin D intake and excess 1,25-dihydroxyvitamin D_3_ production associated with granulomatous diseases. Additional genetic causes of PTH-independent hypercalcemia include Williams syndrome, Down syndrome, blue diaper syndrome, and idiopathic infantile hypercalcemia (1). Finally, while uncommon, hypercalcemia due to excess parathyroid hormone-related protein (PTHrP), independent of PTH, has been reported with malignancy as well as renal dysplasia, including congenital anomalies of the kidney and urinary tract (CAKUT) (2).

PTHrP is homologous in structure and function to PTH. Both hormones have similar roles regarding calcium regulation, including increasing osteoclast activity leading to bone resorption and increased serum calcium levels (2). While PTH functions primarily as an endocrine hormone, PTHrP works in an autocrine and paracrine manner and is found in both fetal and adult tissues including epithelial tissue, endocrine glands, and the central nervous system (3). In the kidney, PTHrP has been implicated in the modulation of tubuloepithelial cell growth and inflammation. Increased expression of PTHrP is associated with renal dysplasia and the development of proteinuria in animal models (4, 5). While cases of infantile hypercalcemia secondary to elevated PTHrP have been reported in individuals with known renal dysplasia, few, if any, reports exist of PTHrP-related hypercalcemia as an initial finding before renal disease and in the absence of malignancy. Here we report a case of hypercalcemia with elevated PTHrP later found to be a precursor to congenital nephrotic syndrome. Written consent from the family was obtained for this report.

## Case Presentation

A 10-month-old female was brought to the pediatric emergency department for failure to thrive without other symptoms. At presentation, her weight-for-height was at the 1st percentile, Z-score −4.25 (weight 5.52 kg, <.01st percentile; length 63.1 cm, <1st percentile). She was born at term after an uncomplicated pregnancy, and weight was appropriate for gestational age (3.40 kg at birth). Birth length was unavailable but noted to be >10th percentile. Her postnatal course was initially unremarkable. Motor development was mildly delayed, as the patient could roll over but was not yet crawling. Broad baseline testing was initiated, and her biochemical findings included elevated serum calcium 14.3 mg/dL (3.58 mmol/L; reference range [RR] 9.0-11.0 mg/dL), normal phosphate 7.0 mg/dL (2.26 mmol/L; RR 4.8-8.4 mg/dL), undetectable PTH <3 pg/mL (<3 ng/L; RR 6-89 pg/mL), subclinical elevation in TSH 5.3221 μIU/mL (5.3221 mIU/L; RR .560-4.000 μIU/mL), normal free T4 1.16 ng/dL (14.9 pmol/L, RR .89-1.70 ng/dL), normal total 25-hydroxy vitamin D 52 ng/mL (129.73 nmol/L; RR 25-80 ng/mL), elevated blood urea nitrogen 24 mg/dL (8.57 mmol/L; RR 4-17 mg/dL), and elevated creatinine .60 mg/dL (53.0 µmol/L; RR .31-.53 mg/dL). Her estimated glomerular filtration rate was 47 mL/min/1.73 m^2^ (normal >65) (6). Renal ultrasound revealed bilateral nephrocalcinosis with no other structural abnormalities. Spot urine calcium-to-creatinine ratio was 1.35 (RR .6-.8). Urine studies were otherwise unremarkable, including negative proteinuria. She was admitted for further inpatient evaluation and treatment.

## Diagnostic Assessment

Following initial testing, PTH-independent causes of hypercalcemia were considered. Bilateral nephrocalcinosis suggested idiopathic hypercalcemia of infancy; however, a normal 1,25-dihydroxyvitamin D_3_ level 11.5 pg/mL (27.6 pmol/L; RR 9.9-79.3 pg/mL) and negative genetic testing (Invitae Nephrolithiasis panel, including testing for *CYP24A1* and *SLC34A1*) excluded this diagnosis. Williams syndrome was considered given hypercalcemia with elevated TSH; however, fluorescent in situ hybridization from peripheral blood for the elastin gene showed a normal pattern of hybridization within band 7q11.23, the gene's normal location. Familial hypocalciuric hypercalcemia was also ruled out given hypercalciuria and nephrocalcinosis. PTHrP level was obtained and was detectable at 23.6 pmol/L (RR <2 pmol/L) (4). A computed tomography chest, abdomen, and pelvis was obtained and showed no evidence of malignancy. Lack of renal structural defects on ultrasound eliminated CAKUT as the cause of elevated PTHrP. Given these findings, the patient received an initial diagnosis of hypercalcemia secondary to idiopathic PTHrP elevation.

## Treatment

Initial treatment included 2 days of intravenous fluids, followed by cessation of vitamin D supplementation and use of low-calcium formula. One week following initial treatment, the serum total calcium improved from 14.3 mg/dL (3.57 mmol/L) to 11.3 mg/dL (2.82 mmol/L) and stabilized. Failure to thrive had resolved within 3 months from admission and initial treatment, with improvement in weight-for-length to the 17th percentile (weight 8.5 kg, height 73.8 cm, Z-score = −.95).

In follow-up with nephrology clinic at age 15 months, her urinalysis showed nephrotic-range proteinuria. Additional findings included elevated urine protein/creatinine ratio 50 (RR .31-.53), low serum total protein 5.2 g/dL (52 g/L; RR 6.1-7.5), and low albumin 2.1 g/dL (.32 mmol/L; RR 3.8-4.7), which was decreased from 2.7 g/dL (.41 mmol/L) 2 months prior. Facial edema was also noted. Given the new development of nephrotic syndrome, minimal-change disease was considered; however, the nonabrupt onset of proteinuria and the patient's young age made this diagnosis unlikely. A genetic defect with podocytes was also considered, and a genetic panel of 42 genes including *WT1* and *PLCE1* (associated with podocyte function) was obtained (CTGT Genetic Testing, Nephrotic Syndrome and Related Disorders) and showed heterogenous changes in *NUP205* (c.281C >G, Ala94Gly, a conservative change unlikely to substantially affect the resultant protein's function, and c.488 + 5T > C causing a base change of unknown significance in the gene's donor splice site) and *FN1* (c.2834A > G, Asn945Ser). These were variants of uncertain significance. Parental genetic testing showed that the mother, who was in good health, carried both *NUP205* variants, indicating their position on the same allele and thus not responsible for autosomal recessive disease. Nonspecific treatment of nephrotic syndrome was prescribed including lisinopril and low-dose diuretics.

## Outcome and Follow-up

**Figure 1.**
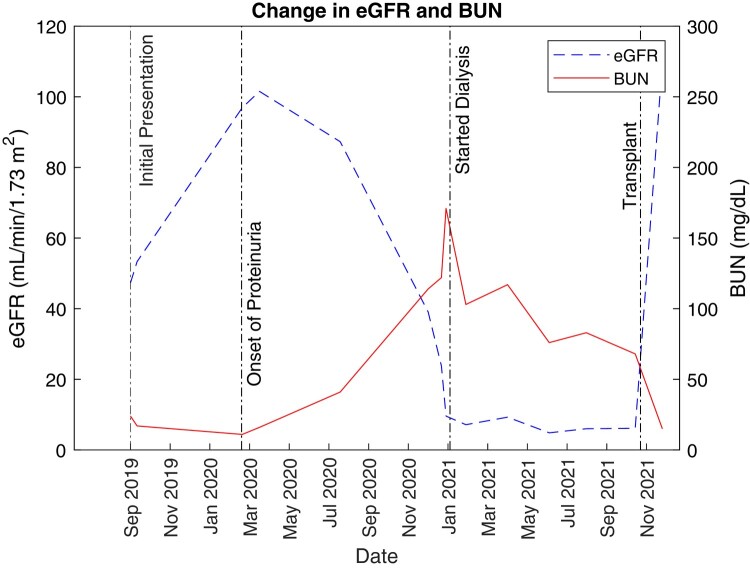
Patient's eGFR and BUN trends over 2 years. Note the precipitous drop in eGFR soon after the onset of proteinuria.

Abbreviations: BUN, blood urea nitrogen; eGFR, estimated glomerular filtration rate.

**Figure 2.**
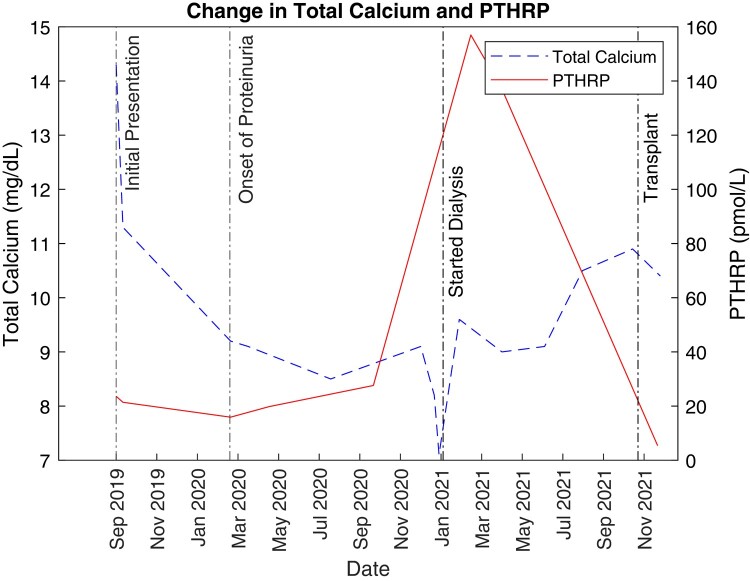
Patient's total calcium and PTHrP trends from initial presentation to transplantation, over 2 years. Note the elevation in PTHrP at presentation (normal <2 pmol/L) prior to onset of detectable proteinuria.

Abbreviations: PTHrP, parathyroid hormone-related protein.

Over the following 8 months from diagnosis, the patient's renal function deteriorated (Fig. 1), and she progressed rapidly from stage 4 chronic kidney disease to stage 5 with uremic symptoms (azotemia, vomiting). Her PTHrP trends showed significantly higher elevation during this time (Fig. 2). She underwent dialysis and subsequently received a living-related donor kidney transplant, leading to symptom resolution including near normalization of her PTHrP level.

## Discussion

To our knowledge, this report is the first to document infantile hypercalcemia secondary to elevated PTHrP without neoplasms or anatomic kidney abnormalities; furthermore, it is the first to detail these findings as a precursor to clinically significant congenital nephrotic syndrome.

PTHrP, a structural analogue to PTH that works in a para- and autocrine manner, increases bone resorption and therefore serum calcium levels via increased osteoclast activity (2). While the exact mechanism of elevated PTHrP in nephrotic syndrome remains unclear, it may be related to PTHrP's role in renal regenerative processes. Data suggest that the protein promotes renal blood flow and glomerular mesangial and tubular epithelial cell regeneration after injury. Experimental studies have shown that PTHrP is important in tubulointerstitial cell survival (via prosurvival mechanisms involving the Runx2 pathway) as well as inducing fibrosis in experimental models of nephropathy (7); more specifically, increased expression of PTHrP has been found in podocytes after murine toxic renal failure and has also been implicated in glomerular healing thereafter (8). PTHrP elevations have been noted in several animal models of nephropathies: studies have shown increased PTHRP mRNA levels in tubuloepithelial cells after ischemic injury in rat kidneys after cyclosporine-induced chronic kidney failure and in a protein-overload rat model of tubulointerstitial nephropathy (5). Elevated PTHrP has also been associated with renal regenerative processes after folate-induced renal damage in rats (7). Therefore, it is possible that renal injury may induce a reactive increase in PTHrP, thereby causing secondary hypercalcemia.

This mechanism may explain the patient's novel presentation of hypercalcemia and elevated PTHrP in the absence of appreciable renal abnormalities prior to the development of congenital nephrotic syndrome. Given the uncertain etiology of the patient's congenital nephrotic syndrome, the genetic testing is of particular interest. While the change in *NUP205* here was previously unreported, *NUP205* mutations are associated with podocyte membrane dysfunction and nephrotic syndrome; however, NUP205-related nephrotic syndrome is typically inherited in an autosomal recessive manner, making it a less likely cause for our patient's presentation (9). The *FN1* variant was also previously unreported; however, while *FN1* mutations can cause autosomal-dominant glomerulopathy with fibronectin deposits via changes in podocyte membranes, this disorder often presents more gradually and in older individuals (20-40 years) (10). If the patient's nephrotic syndrome was due to a mutation affecting renal podocytes, it is possible that PTHrP upregulation from damaged cells preceded clinically measurable nephrotic syndrome. This would account for PTHrP elevation and subsequent hypercalcemia, with an absence of detectable renal anomalies early in the patient's disease course, while continued renal cell apoptosis would eventually lead to detectable changes in estimated glomerular filtration rate and blood urea nitrogen.

In pediatric patients, elevated levels of PTHrP have been associated with neoplasms including giant hepatoblastoma, ovarian dysgerminoma, pheochromocytoma, mesonephric blastoma, and metanephric adenoma (2). However, to the authors' knowledge, only 2 other reports currently detail elevated PTHrP in the setting of congenital abnormalities of the kidneys (excluding neoplasm): in CAKUT (4) and in multicystic dysplastic kidney (2). In these studies, anatomical kidney abnormalities were known prior to the development of PTHrP-induced hypercalcemia. We are unable to find prior reports of elevated PTHrP associated with renal function abnormalities in a pediatric patient with anatomically normal kidneys. The findings in our patient suggest that elevated PTHrP may have been a precursor to progressive congenital nephrotic syndrome.

In the absence of other explanations for hypercalcemia due to PTHrP elevation, awareness of the connection between PTHrP's role in renal cellular damage and regeneration, including congenital nephrotic syndrome, may allow clinicians to more closely monitor for and anticipate changes in patients' renal function.

## Learning Points

- When an infant presents with hypercalcemia, initial testing should include evaluation of PTH, inactive and active forms of vitamin D, urine studies, and PTHrP.
- In settings of elevated PTHrP, computed tomography for both malignancy and renal/urinary ultrasound are warranted.
- In infants and children with hypercalcemia secondary to elevated PTHrP and no malignancy or anomalies of the renal/urinary system, close monitoring of renal function may be indicated for early identification of progressive renal disease, including congenital nephrotic syndromes.

## Data Availability

Data sharing is not applicable to this article as no datasets were generated or analyzed during the current study.
